# Knowledge and Awareness of Parents About Pediatric Obstructive Sleep Apnea in Jeddah: A Cross-Sectional Study

**DOI:** 10.7759/cureus.38960

**Published:** 2023-05-13

**Authors:** Rayan M Alosaimi, Gutaybah Alqarni, Mohammed T Musslem, Feras F Filfilan, Essa A Alazmi, Jehad R Alsaedi, Talal Y Alghamdi, Hosam Amoodi

**Affiliations:** 1 Medicine, University of Jeddah, Jeddah, SAU; 2 Otolaryngology-Head and Neck Surgery, University of Jeddah, Jeddah, SAU; 3 Otolaryngology-Head and Neck Surgery, Dr. Soliman Fakeeh Hospital, Jeddah, SAU

**Keywords:** pediatric obstructive sleep apnea, knowledge and awareness, jeddah saudi arabia, a cross-sectional study, sleep-disordered breathing

## Abstract

Background

Obstructive sleep apnea (OSA) is relatively common in childhood and is characterized by repeated partial or complete blockages of the upper airway during sleep. Children affected by OSA can experience various symptoms including snoring, restless sleep, and behavioral issues such as hyperactivity, impulsivity, and aggression, which interfere with their quality of life. Additionally, OSA can result in serious conditions such as cardiovascular and metabolic diseases. This study aims to determine the level of knowledge and awareness of OSA among parents in the Jeddah region.

Methodology

An observational cross-sectional study was conducted to determine the level of awareness of OSA among all parents in Jeddah, Saudi Arabia. Participants were recruited through social media platforms. The online survey assessed the knowledge regarding the OSA definition, risk factors, associated symptoms, and treatment.

Results

A total of 462 participants were included. Only 16% of participants had a good level of knowledge of OSA, while the remaining 84% had a poor level of knowledge. The mean knowledge score was 15.39 ± 5.8, with a significant difference between occupations (P=0.039).

Conclusion

Our study reveals that parents in Jeddah, Saudi Arabia have a low level of knowledge about pediatric OSA, with only 16% demonstrating good knowledge and less than half recognizing the definition of OSA. This lack of knowledge could lead to delays in diagnosis and treatment, negatively impacting children's health and academic performance. Common symptoms of OSA reported by parents were restless sleep, mouth breathing, and snoring, but bedwetting and hyperactivity were poorly recognized. Adenoids, allergic sinusitis, enlarged tonsils, asthma, and obesity were identified as risk factors for OSA. Improving parental awareness of OSA through public campaigns, doctor consultations, and education programs is crucial. Further studies are needed to assess the effectiveness of these interventions.

## Introduction

Obstructive Sleep Apnea (OSA) is a sleep-related breathing disorder characterized by repeated partial or complete blockages of the upper airway during sleep. These blockages lead to disruptions in normal sleep patterns and breathing, which result in sleep fragmentation and occasional episodes of hypoxia and hypercapnia [1,2]. If left untreated, OSA can negatively affect the quality of life and normal sleep and lead to several consequences [2].

OSA is a relatively common condition in childhood, with a prevalence rate between 1% to 5%. Risk factors for OSA include adenotonsillar hypertrophy and obesity [3]. Symptoms of OSA can include snoring, restless sleep, excessive daytime sleepiness, inattention, learning difficulties, and behavioral problems such as hyperactivity, impulsiveness, defiance, and aggression, which can sometimes lead to a diagnosis of attention deficit hyperactivity disorder (ADHD) [2-4]. While OSA is treatable, failure to address the condition can lead to severe consequences such as cardiovascular, neurocognitive, and metabolic diseases, ultimately affecting the child's quality of life [2,3].

Limited research has been conducted on pediatric OSA in Saudi Arabia. Existing studies consistently demonstrate low awareness among the general population and medical personnel. A study conducted in the Asir region of Saudi Arabia found poor awareness of all aspects of OSA among its population, with most subjects lacking sources of knowledge about the condition [5]. Similarly, another study among primary care physicians in Saudi Arabia revealed a lack of knowledge about sleep medicine and an unrecognition of the importance and impact of sleep disorders [6]. In addition, a study on parents' knowledge and awareness of OSA in Saudi Arabia found that a third of the population had a low level of knowledge [7]. A study conducted in Jeddah, Saudi Arabia assessed the knowledge and awareness of 146 parents who visited a general pediatric clinic, regarding pediatric OSA. The study revealed a lack of knowledge among parents about pediatric OSA. [8]. These findings highlight the critical need for increased education and awareness campaigns among medical professionals and the general public regarding pediatric OSA in Saudi Arabia.

This study aims to assess the level of knowledge and awareness of OSA among parents in Jeddah and identify potential factors associated with their knowledge level.

## Materials and methods

Design, participants, and setting

We conducted a descriptive cross-sectional study from January 2023 to March 2023. In this study, we included all parents above 18 years old living in Jeddah. We excluded all participants who didn’t have children and all responses outside Jeddah. Dr. Soliman Fakeeh Hospital Scientific Research Review Committee approved the study.

Questionnaire

With permission, we used a validated and published questionnaire [7]. The questionnaire was edited and revalidated by an expert statistician who conducted a pilot study of 24 participants. The Cronbach's alpha value was 0.82. The questionnaire is composed of two parts. The first part contained eight sociodemographic questions, including age, nationality, gender, marital status, education, occupation, number of children, and whether the participant had children affected with OSA. The second part contained questions assessing the knowledge regarding the definition, risk factors, symptoms, and treatment of OSA, in addition to questions about the information source and the recommended method of raising awareness. Each question in the second part was given a weight based on its difficulty or importance, and the total knowledge score was calculated by adding up the weights for all correct answers. The maximum score was 30. Participants were divided into two groups based on their knowledge level: good knowledge (>70%) and poor knowledge (≤70%).

Data collection, processing, and analysis

The electronic survey was created using Google Forms (Google LLC, Mountain View, CA, USA). Using the snowball sampling technique, social media was employed to reach our target sample, and parents were encouraged to share the survey. Categorical data were presented as frequencies and percentages in tables and figures, whereas continuous data were presented as means and standard deviation. To assess the variation in knowledge scores across different sociodemographic characteristics, a one-way analysis of variance (ANOVA) was conducted. With a 95% confidence interval, statistical significance was defined as a p-value ≤ 0.05. Data were managed and analyzed using Microsoft Office Excel (Microsoft Corp., Redmond, WA, USA) and SPSS version 29 (IBM Corp., Armonk, NY, USA).

Ethical consideration

The study was approved by the Institutional Review Board at Dr. Soliman Fakeeh Hospital in Jeddah, Saudi Arabia (Approval No. 396/IRB/2023). The questionnaire started with a written consent agreement indicating their agreement to participate in the study by submitting the questionnaire.

## Results

Table 1 provides a summary of the sociodemographic characteristics and mean knowledge scores of the participants, The largest age group was between 41 to 50 years old (34.4%). The majority of the participants were married Saudi parents, and 72.9% of them were female. Furthermore, a large proportion of the participants held a bachelor's degree. Over half of the participants reported having one to three children (53.7%). The mean knowledge score of the participants was 15.39 ± 5.8. A one-way analysis of variance (ANOVA) revealed a statistically significant difference in mean knowledge scores across occupations (P = 0.039). No other statistically significant difference was found between sociodemographics and knowledge scores.

**Table 1:** Sociodemographic characteristics and mean knowledge score by sociodemographic variables.

Characteristics		Number of participants (%)	Knowledge score (mean ± standard deviation)	p-value
Gender	Male	125 (27.1)	15.25 ± 6.43	.756
	Female	337 (72.9)	15.44 ± 5.61	
Age group (years)	18-30	59 (12.8)	17.15 ± 5.44	.090
	31-40	150 (32.5)	15.12 ± 5.67	
	41-50	159 (34.4)	15.51 ± 6.08	
	51-60	72 (15.6)	14.38 ± 5.87	
	60+	22 (4.8)	14.88 ± 5.52	
Nationality	Saudi	433 (93.7)	15.42 ± 5.90	.627
	Non-Saudi	29 (6.3)	14.87 ± 4.87	
Marital status	Married	428 (92.6)	15.38 ± 5.80	.969
	Divorced	25 (5.4)	15.26 ± 6.14	
	Widow	9 (1.9)	15.83 ± 7.50	
Education	Uneducated	2 (0.4)	15.50 ± 10.60	.097
	High school	84 (18.2)	13.95 ± 5.80	
	Undergraduate	28 (6.1)	16.10 ± 5.91	
	Higher education	80 (17.3)	16.37 ± 6.05	
	Bachelor	268 (58)	15.47 ± 5.71	
Occupation	Health sector employee	49 (10.6)	17.87 ± 6.98	.039
	Engineer	20 (4.3)	13.57 ± 5.60	
	Teacher	117 (25.3)	15.17 ± 5.44	
	Soldier	9 (1.9)	12.94 ± 8.23	
	Pilot	2 (0.4)	16 ± 1.41	
	Lawyer	3 (0.6)	13 ± 4.09	
	Other	262 (56.7)	15.26 ± 5.64	
Number of children	1-3	248 (53.7)	15.46 ± 5.91	.348
	4-7	202 (43.7)	15.16 ± 5.73	
	More than 8	12 (2.6)	17.62 ± 5.96	
Knowledge score		462 (100)	15.39 ± 5.84	

The primary sources of information about OSA among parents were the Internet and social media (36.8%), followed by medical articles (23.6%), and individuals affected by OSA (21.6%). However, a notable proportion of parents (35.9%) reported being unfamiliar with the condition. Additionally, 22.1% of the parents stated that they had at least one child suffering from OSA. Nearly half of the parents (49.8%) correctly identified "repeated episodes of obstruction of breathing during sleep" as the definition of OSA. However, a significant proportion (40.5%) of parents reported that they did not know about OSA.

A large percentage agreed that “early intervention and management can reduce complications” and “parents' awareness of OSA can help in reducing the burden” (88.1%). However, a considerable percentage (63%) answered incorrectly when asked about the relationship between OSA and depression and the role of genetic factors in causing OSA, as shown in Table 2.

**Table 2:** Percentages and frequencies of general knowledge answers

General knowledge items		Frequency	Percentage
Do you know that childhood obstructive sleep apnea can affect a child’s school performance?	No	247	53.5
	Yes	215	46.5
Do you know that children with obstructive sleep apnea have a higher prevalence of depression than other children?	No	293	63.4
	Yes	169	36.6
Do you know that childhood obstructive sleep apnea affects attention and behavior?	No	243	52.6
	Yes	219	47.4
Do you think that the genetic factor can have a role in the cause of childhood obstructive sleep apnea?	No	291	63.0
	Yes	171	37.0
Do you think that childhood obstructive sleep apnea can be managed?	No	63	13.6
	Yes	399	86.4
Do you think early intervention and management can reduce the possible risk of complications in children with obstructive sleep apnea?	No	55	11.9
	Yes	407	88.1
Do you think parents' awareness about childhood obstructive sleep apnea can help reduce the burden on them and the population?	No	55	11.9
	Yes	407	88.1

**Figure 1:**
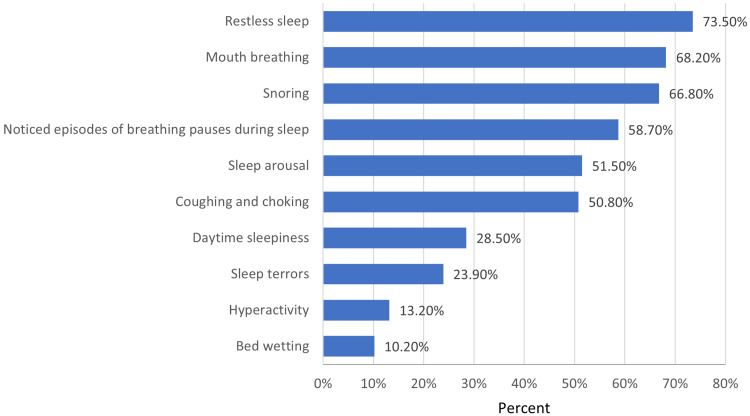
The percentages of correct answers regarding the symptoms of childhood OSA

**Figure 2:**
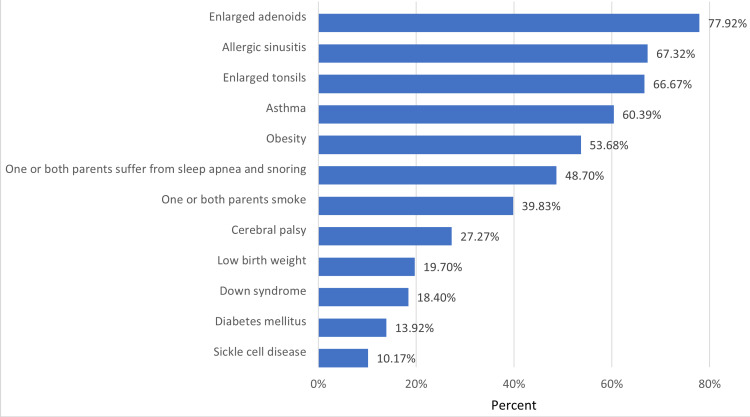
The percentages of correct answers regarding the risk factors of childhood OSA

Regarding OSA symptoms, the most commonly reported symptoms were restless sleep, followed by mouth breathing and snoring, as demonstrated in Figure 1. Figure 2 shows the results of parents' knowledge of OSA risk factors, indicating that the majority of participants correctly identified adenoids, allergic sinusitis, enlarged tonsils, and asthma as risk factors for OSA. In contrast, sickle cell anemia and diabetes mellitus were identified as risk factors by only a small proportion of respondents, ranging from 10.17% to 13.92%. Table 3 shows that based on their total knowledge scores, 84% of parents had a poor level of knowledge of pediatric OSA, while the remaining 16% had a good level of knowledge.

**Table 3:** Frequencies and percentage of knowledge level

Item	Frequency	Percentage
Poor knowledge	388	84%
Good knowledge	74	16%
Total	462	100%

When asked about the most effective ways to increase awareness about OSA, 35.1% of respondents believed that consulting a doctor was the best method. Volunteer awareness campaigns were recommended by 29.3% of respondents, while 35.7% thought that the internet and social media were the most effective method.

## Discussion

Healthy sleep is crucial for normal child development, and OSA is a common condition affecting up to 5% of children [3,9]. As parents play a vital role in managing OSA, their awareness and recognition of the condition can significantly improve the quality of life of affected children [3,9]. Therefore, we believe that it is critical to increase parental awareness of OSA to ensure that children can have healthy and normal lives. Despite the importance of parental awareness, only two studies have been conducted in Saudi Arabia to assess it [7,8]. This study aimed to evaluate the knowledge and awareness of OSA among parents in Jeddah, Saudi Arabia.

Our study included 462 participants and indicated a widespread lack of knowledge about OSA, with only 16% of parents demonstrating a good level of knowledge, and a mean knowledge score of 15.39 ± 5.84. Mothers who comprised the majority of our sample (72.9%), had a similar level of knowledge as fathers, While there was a significant difference in knowledge scores among parents with different occupations (p = 0.039), we did not observe any other significant differences based on sociodemographic factors.

In our study less than half of the participants correctly recognized the definition of OSA, which is consistent with the findings of Alosaimi et al. and Xu et al.'s studies [8,10], but contrary to the results of Bashir et al.'s study, where a large percentage responded correctly [7]. Moreover, our findings revealed that most of our participants did not agree that OSA can impact educational performance, which is similar to the results of Alosaimi et al.'s study and a study conducted among medical and dental students and fresh graduates (interns) [8,11]. These results highlight the need for increased efforts to raise awareness about the definition of OSA and its potential impacts on children's health and academic performance.

In our study, we found that restless sleep, mouth breathing, and snoring were the most reported symptoms. These findings are consistent with previous studies by Alosaimi et al. and Bashir et al. However, our study showed a significantly higher correct response rate compared to Bashir et al.'s study [7,8]. Our findings also revealed that bedwetting and hyperactivity were poorly recognized by the parents, similar to the results of studies conducted by Alosaimi et al. and Xu et al. [8,10]. Similarly, a recent study conducted by DiNardo et al. revealed that parents had a mean score of 6.3 out of 13 for recognizing pediatric OSA symptoms, with nocturnal enuresis and hyperactivity being the least recognized symptoms by 27% and 37% of parents, respectively. The study also showed that parents who expressed greater concern about OSA had a higher cumulative knowledge score [12]. These results confirm our findings that parents may not be aware of the full range of symptoms associated with OSA, highlighting the importance of educating parents about the full range of symptoms associated with OSA to ensure timely diagnosis and treatment.

The present study found that most participants agreed that adenoids, allergic sinusitis, enlarged tonsils, asthma, and obesity are risk factors for OSA, consistent with the findings of previous studies by Alosaimi et al. and Bashir et al. However, the recognition of obesity as a risk factor was poor in the Bashir et al. study, whereas in our study, it was well-recognized [7,8]. Furthermore, the least recognized risk factors were sickle cell disease, diabetes mellitus, Down syndrome, low birth weight, and cerebral palsy. These findings were consistent with the Alosaimi et al. study but contradicted the findings of the Bashir et al. study, which reported better recognition of these risk factors [7,8]. One possible explanation for this discrepancy is that our study and the Alosaimi et al. study were both conducted in Jeddah, while the Bashir et al. study was conducted across Saudi Arabia [7,8]. These results highlight the need for targeted education and awareness campaigns among parents in Jeddah regarding the full range of risk factors associated with pediatric OSA.

According to the participants' responses, doctor consultations, internet/social media, and volunteer campaigns are nearly equally effective approaches for raising awareness about childhood OSA. Our findings indicate that there is a need to increase awareness about OSA among parents, given the significant impact that their knowledge can have on diagnosis and treatment. Healthcare professionals can play a crucial role in raising awareness about the disease. Social media and the internet can also be valuable tools for reaching a wider audience. These results should be considered in the development of future awareness campaigns, and decision-makers should make every effort to educate parents effectively about the risks and symptoms of OSA in children.

While our study represents an important contribution to understanding parental awareness of childhood OSA in Jeddah, it is important to acknowledge its limitations. The survey-based methodology used in our study may have introduced recall bias, which could have affected the accuracy of our results. In addition, some participants may have relied on online resources to answer the survey questions, which may have further influenced the validity of our findings. Furthermore, our sample was not evenly distributed across specific sociodemographic characteristics, which may limit the generalizability of our findings. Future studies should examine parental awareness of OSA in other regions of Saudi Arabia.

## Conclusions

Our study highlights the low level of knowledge and awareness of pediatric OSA among parents in Jeddah, Saudi Arabia. Despite parents' critical role in managing OSA, only 16% of participants demonstrated a good level of knowledge, and less than half correctly recognized the definition of OSA. This lack of knowledge could lead to delays in diagnosis and treatment and have negative impacts on children's health and academic performance. The study also found that the most common symptoms of OSA reported by parents were restless sleep, mouth breathing, and snoring. However, bedwetting and hyperactivity were poorly recognized by parents as symptoms of OSA. Adenoids, allergic sinusitis, enlarged tonsils, asthma, and obesity were identified by most participants as risk factors for OSA. Given the importance of parental awareness in managing OSA, efforts should be made to improve parental knowledge and awareness of this condition. This can be achieved through various means, such as public awareness campaigns, doctor consultations, and education programs. Further studies are needed to assess the effectiveness of these interventions in increasing parental awareness and improving the management of pediatric OSA.
